# Spatial variation in risk factors for anti-hepatitis E antibody titers in a population-based German study

**DOI:** 10.1038/s41598-025-26850-z

**Published:** 2025-11-21

**Authors:** Andrea C. Díaz, Till Ittermann, Matthias Nauck, Astrid Petersmann, Henry Völzke, Birgit Schauer

**Affiliations:** 1 Department SHIP-KEF, Institute of Community Medicine, University Medicine Greifswald, Greifswald, Germany; 2 Institute for Medical Information Processing, Biometry and Epidemiology - IBE, LMU Munich, Munich, Germany; 3 Pettenkofer School of Public Health, Munich, Germany; 4 Institute of Clinical Chemistry and Laboratory Medicine, University Medicine Greifswald, Greifswald, Germany; 5 Institute of Clinical Chemistry and Laboratory Medicine, University Medicine Oldenburg, Oldenburg, Germany; 6 Present Address: Department of Infection and Immunity, Luxembourg Institute of Health, Esch-sur-Alzette, Luxembourg

**Keywords:** Companion animals, Germany, Hepatitis E, Risk factors, Serology, Risk factors, Infectious diseases

## Abstract

The transmission of hepatitis E virus (HEV) genotype 3 remains unclear. Up to date, it is predominantly considered a foodborne disease. However, there is growing evidence that other transmission pathways exist. The aim of this study was to analyze risk factors for anti-HEV IgG titers in the Study of Health in Pomerania, a population-based cohort located in Northeast Germany. We performed cross-sectional analyses using quantile and logistic regression. Spatial location was included as an interaction term to explore whether residential location of the participant acts as an effect modifier. The risk factors assessed were consumption frequency of meat, sausage, fish and raw vegetables, high-risk occupation and animal contact. The estimated seroprevalence of anti-HEV IgG was 27.3% (95% confidence interval [CI] 26.0%, 28.6%). Participants living in rural coastal areas had 14.5 U/ml higher antibody titers both for high meat (95% CI 3.9, 25.2; *P* = 0.008) and high sausage consumption (95% CI 4.1, 24.9; *P* = 0.006) in comparison with low consumption. In urban areas, participants who had contact with domestic carnivores had higher antibody titers (by 12.8 U/ml; 95% CI 0.3, 25.3; *P* = 0.046). Our results imply that risk factors of HEV exposure might differ among geographical regions. More research is needed to clarify the mechanisms underlying these findings.

**Supplementary Information:**

The online version contains supplementary material available at 10.1038/s41598-025-26850-z.

## Introduction

In the European Union, the number of notified hepatitis E virus (HEV) infections increased in a stepwise manner between 2005 and 2015 with a total of 21,018 confirmed cases observed over this period^1^. Eighty percent of these cases were reported in Germany, France and the UK. Autochthonous cases in Europe are mostly caused by genotype 3. In Germany estimates for anti-HEV IgG seroprevalence, ranged from 2.6% to 29.5% in different regions and populations^2^.The estimation of true seroprevalence for HEV is challenging, because there is no gold standard for the detection of anti-HEV IgG/IgM in serum^3^. Therefore, serological results are dependent on the commercial test used^4^.

Humans are susceptible to five of the eight HEV genotypes, and these belong to genus *Orthohepevirus* A, family *Hepeviridae*^5^. Genotypes 1 and 2 are obligate human pathogens. Genotypes 3, 4 and 7, are also found in pigs, wild boar (*Sus Scrofa*), rabbits (*Oryctolagus cuniculus*) and goats among other species^5^. Wild boar and domestic pigs are considered the main reservoir of the virus in several countries including Germany^6,7^. In addition, a variety of species are also susceptible to the virus such as foxes (*Vulpes vulpes*), red (*Cervus elaphus*) and roe deer (*Capreolus capreolus)*, and raccoon dogs (*Nyctereut es procyonoides*)^8–10^.

Zoonotic transmission through food is considered the main source of HEV genotype 3 infection, especially through undercooked pork and wild boar products^6^. HEV genotype 3 RNA has also been detected in shellfish, raw vegetables and fruit^6,11^. Shellfish, pork and game meat (boar) have been directly linked to HEV outbreaks^12–14^. However, fruit and vegetables have not been identified as an effective transmission vehicle^6,12^. Other potential sources of HEV transmission to humans are direct contact with susceptible species, indirect contact (feces) or consumption of other animal proteins such as deer^15^. Additionally, evidence suggests that domestic carnivores such as dogs and cats may play a role in the epidemiology of the disease. In Germany, a previous study found an anti-HEV IgG/IgM seroprevalence of 32.3% (95% CI 20.9, 43.68) and 56.6% (95% CI 46.0, 67.3) in cats and dogs, respectively^9^. Possible pathways for human-to-human transmission are through contact with blood (incl. transfusion) and through solid organ transplants^1,16–18^.

Mecklenburg-Pomerania is a region of interest for HEV research in Germany due to a higher incidence rate compared to other regions^19^. Using data from a cohort of the Study of Health in Pomerania, we aimed to detect associations between risk factors such as meat consumption and anti-HEV IgG titers and to investigate the impact of spatial location on our risk factors of interest. To the authors’ knowledge, no population-based research has been conducted investigating effect modification of risk factors for anti-HEV IgG due to spatial location.

## Methods

### Study region and sample size

The Study of Health in Pomerania (SHIP) is located in Mecklenburg-Pomerania, Northeast Germany. The study region includes two districts, West Pomerania-Rügen and West Pomerania-Greifswald excluding the islands ^20^. SHIP is constituted by three cohorts, START (since 1997), TREND (since 2008) and NEXT (since 2021). For SHIP-TREND, a population-based random sample of adults (20–79 years of age) residing in the study region was drawn from population registries. Written informed consents were provided by all participants of SHIP according to the principles of the Declaration of Helsinki. The studies were approved by the Ethics Committee at the University Medicine Greifswald, Germany (approval number BB 39/08). Details on the study design have been reported previously^20,21^. We used data from TREND, a cohort composed of two examinations. The baseline, TREND-0 (2008–2012), included 4,420 participants and the first follow-up, TREND-1 (2016–2019), 2,507 participants. We excluded participants without HEV serology results. Animal husbandry information was only collected in TREND-1. Therefore, we only included individuals that participated in both examinations of TREND for the analyses investigating animal contact.

### Assessments

Data were collected through questionnaires and blood specimens. Animal husbandry information was collected in TREND-1 only. The rest of the data were collected in TREND-0.

For animal contact, we included the question “Have you owned one of the mentioned animal species during the last 10 years?”. Results were analyzed by animal groups to avoid sparse data. Dogs and cats were grouped into domestic carnivores and horses, cows, pigs and goats into farm animals.

Frequency of food consumption was measured with the nutritional scale validated by Winkler et. al.^22^. The variables were transformed into binary variables to facilitate interpretation. High risk occupations were grouped into one binary variable that allocated the participant in a high-risk/low-risk group. High risk occupations considered included: medical doctor, veterinary doctor, nurses, abattoir workers and jobs involving contact with raw meat or raw fish, farmers, foresters or gardeners. The food groups analyzed were meat, sausage, raw vegetables and fish.

Spatial location of residence was derived from the postcode and city/town of the participant’s postal address. Four different categorical variables were generated to assess the effect of spatial location by allocating the district of residence to the following categories: inland/coast, urban/urban associated/rural, urban/rural coastal/rural inland and rural/urban (combining urban associated with urban locations). The purpose behind the variety of the spatial variables was to preserve the granularity of the information while protecting the privacy of the participants.

Blood samples were drawn from the cubital vein. Serum aliquots analyzed were stored at − 80 °C until testing. The commercial test used was HEV IgG enzyme-linked immunosorbent assay (ELISA), Mikrogen RecomWell, Neuried, Germany. Serological assays to determine anti HEV-IgG antibodies lack estimations of true sensitivity and true specificity due to the lack of a gold standard test. Mikrogen estimated sensitivity and specificity as 100% (six samples) and 98.5% (135 samples) ^23^. In the absence of a gold standard, a study comparing three HEV IgG assays (Mikrogen RecomWell, Wantai and Euroimmun) assumed a positive result (designated “overall HEV IgG”) as likely when at least two of the three IgG assays yielded a positive test result. RecomWell showed the highest Cohens Kappa correlation to the overall result (Kappa = 0.93) compared with the other two assays ^3^. Laboratory analysis was performed at the Institute of Clinical Chemistry and Laboratory Medicine, University Medicine Greifswald, Germany.

### Statistical analysis

Prevalence was estimated using the cut-off points recommended by the manufacturer. Borderline results were classified as negative. Subsequently, we conducted a two-stage cross-sectional analysis. First, we used logistic regression models to determine associations between potential risk factors and HEV status. Second, we used quantile regression models to assess associations between the risk factors and anti-HEV IgG titers. The 80^th^ percentile was used because of the skewed distribution of the outcome and the proximity to the cut-off value of the test to this percentile (> 24 U/ml). At both stages, separate regression models were computed for each risk factor. This allowed assessment of its association with the outcome independently of other factors and maintained simplicity in models containing interaction terms. All models were adjusted for sex and age (in years) to control for confounding. Subsequently, we used quantile regression models to investigate the potential effect modification of spatial location on the associations. For this, we introduced interaction terms of spatial location with the respective potential risk factor into the models. The four variables describing the residential location of the participants were introduced as interaction terms in separate models.

A complete-record analysis was conducted since missing data represented less than 5% of the sample ^24^. Standard errors (SE) in quantile regression models were computed using a bootstrap method due to instability of estimates when computing the default method (Huber sandwich when n > 1001) ^25^. Two-tailed P values were estimated using the Wald *t* test for quantile regression and Wald *z* test for logistic regression, statistical significance was defined as a P value of less than 0.05. We performed a sensitivity analysis to assess the robustness of our findings.

As sensitivity analyses, we used a sequence of percentiles (from 10 to 90^th^ by 10) to assess the stability of the estimates resulting from quantile regression. Likewise, for logistic regression we classified borderline results as positive.

Most of the calculations were performed in R Studio V 2022.02.1 using the packages “quantreg”, “epiR” and “marginaleffects”^25–27^. Additionally, STATA V 16.1 (Stata-Corporation, College Station, TX, USA) was used for data cleaning (only for high risk occupation). We report our findings following the STROBE guidelines for observational studies^28^.

## Results

### Seroprevalence

**Fig. 1:**
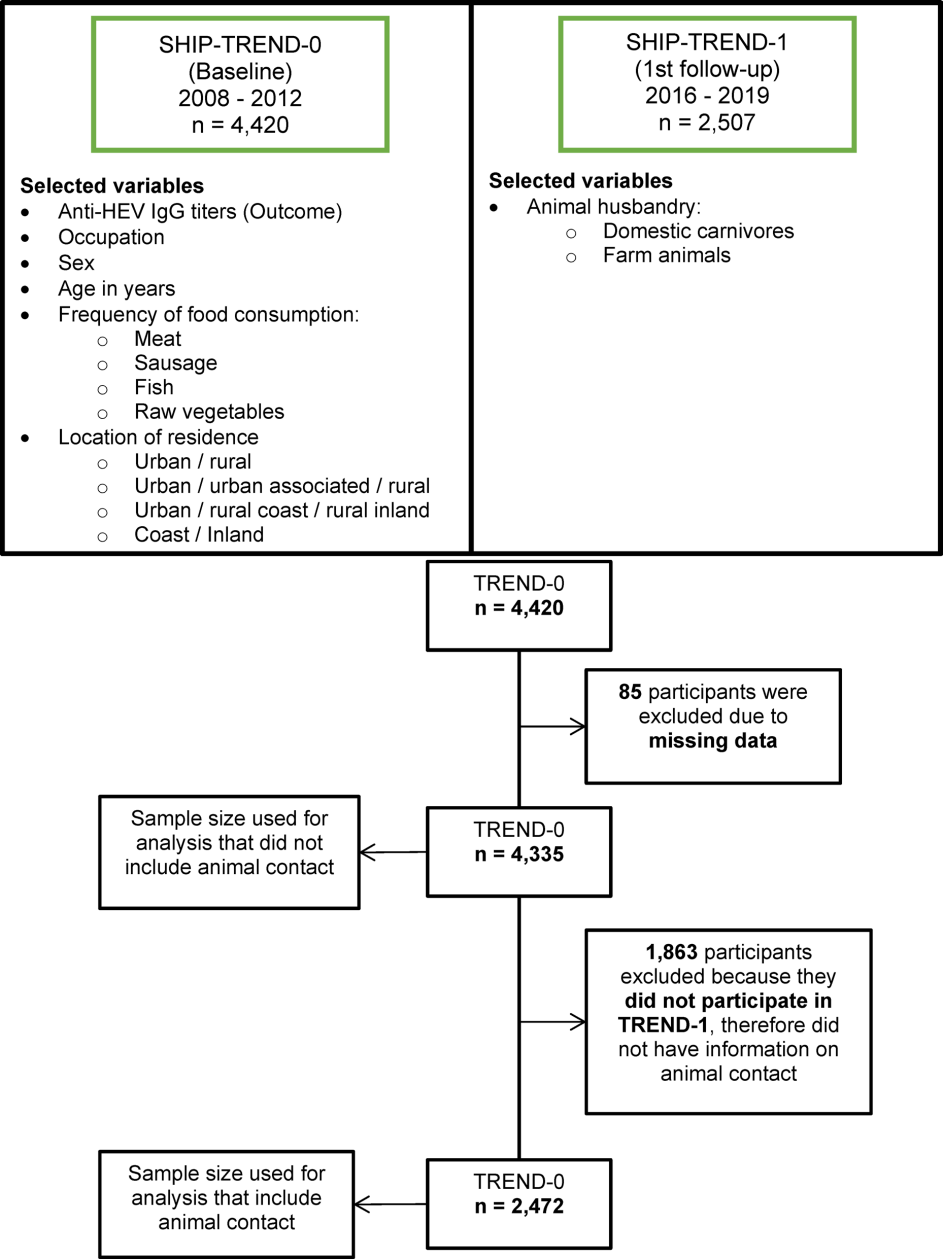
The first section describes sample sizes and time frame of TREND-0 and TREND1 and the variables selected from each round. The second section describes the sample size included for the analysis. Sample size differs for animal contact analyses, because the information of animal contact was only collected in TREND-1.

From the present analysis we excluded all participants from TREND-0 without HEV serological results (n = 85), resulting in a sample size of 4335 participants. Additionally, for animal contact analysis we excluded individuals of TREND-0 that did not participate in TREND-1 as no information on animal contact was available. The resulting sample size for animal contact analysis was 2472 participants (Fig. 1)**.** At baseline, participants had a median age of 53 years (IQR 40—64 years) and 48.6% were male. The estimated seroprevalence was 27.3% (95% CI 26.0, 28.6%). Seroprevalence for investigated risk factor variables and 80^th^ percentiles of anti-HEV IgG titers in each category can be found in Table 1. When stratifying by age, the highest prevalence was in the age groups 60–69 (37.4%; 95% CI 34.1, 40.7%) and 70–79 years (32.7%; 95% CI 28.9, 36.6%), both exceeding significantly the overall estimate (Table 2).

**Table 1:** Prevalence of risk factors for anti-hepatitis E virus (HEV) IgG antibody titers in the total sample and amongst HEV positive individuals (HEV+) as well as 80^th^ percentile of anti-HEV IgG antibodies in the SHIP-TREND-0 cohort in Northeast Germany, 2008–2012 (n = 4335**).**

Risk factor	Category	Total		HEV+		Anti-HEV IgG 80^th^ percentile U/ml (95% CI) ^†^
		n	%	n	%	
Sex	Male	2106	48.6	588	27.9	38.6
	Female	2229	51.4	594	26.7	41.2
Occupation	High risk	533	12.3	154	29.0	41.2
	Low risk	3802	87.7	1028	27.0	39.1
Spatial location						
Coast	Yes	2992	69.0	830	27.7	39.1
	No	1343	31.0	352	26.2	40.0
Rural	Yes	1400	32.3	383	27.4	39.9
	No	2935	67.7	799	27.2	39.1
Urban	Urban	2515	58.0	680	27.0	37.7
	Urban associated	420	9.7	119	28.3	51.5
	Rural	1400	32.3	383	27.4	39.9
Rural/inland	Urban	2935	67.7	799	27.2	39.1
	Rural coast	480	11.1	130	27.1	36.9
	Rural inland	920	21.2	253	27.5	41.3
Frequency of food consumption						
Meat	Low frequency	1380	31.8	383	27.8	42.5
	High frequency	2818	65.0	746	26.5	37.6
Sausage (Chicken and pork)	Low frequency	857	19.8	229	26.7	38.5
	High frequency	3342	77.1	901	27.0	39.1
Fish	Low frequency	3265	75.3	862	26.4	38.1
	High frequency	934	21.6	268	28.7	41.0
Raw vegetables	Low frequency	1737	40.1	480	27.6	41.9
	High frequency	2460	56.8	650	26.4	37.0
Animal contact^‡^						
Domestic carnivores	Yes	1160	46.9	300	25.9	40.2
	No	1309	51.0	367	28.0	38.0
Farm animals	Yes	116	4.7	24	20.7	25.3
	No	2347	94.9	642	27.4	39.3

^†^80^th^ percentile of anti-HEV IgG titers in each category.

^‡^Animal contact analysis was performed on a subset of the baseline sample. Only participants who participated in the TREND-1 examination were included (n = 2472).

Percentages in the Total column are calculated column-wise (total: n = 4335), whilst those in the HEV+ column are calculated row-wise (total: n in the Total column).

**Table 2:** Hepatitis E virus seroprevalence and 95% confidence interval stratified by age in the SHIP-TREND-0 cohort in Northeast Germany, 2008–2012 (n = 4335).

Age range (years)	N	Estimated seroprevalence		Anti-HEV IgG 80^th^ percentile (U/ml) ^†^
		%	95% CI	
20–29	370	11.4	8.3, 15.0	4.3
30–39	684	15.8	13.1, 18.7	10.2
40–49	858	25.9	23.0, 28.9	38.6
50–59	919	31.2	28.2, 34.3	49.3
60–69^†^	830	37.4	34.1, 40.7	52.5
70–79^†^	600	32.7	28.9, 36.6	43.4
≥ 80	74	23.0	14.0, 34.2	40.8
Total	4335	27.3	26.0, 28.6	39.3

^†^Age strata with non-overlapping confidence intervals in relation to total prevalence.

### Logistic regression results

Age in years was associated with HEV status in all logistic regression models (OR 1.02; 95% CI 1.02, 1.03; *P* < 0.001), whilst neither sex nor any of the other investigated risk factors assessed were significantly associated (Table 3).

**Table 3:** Logistic regression analysis of risk factors for anti-hepatitis E IgG titers in the SHIPTREND cohort in Northeast Germany, 2008–2012 (n = 4335).

Risk factor	Category	Logistic regression		*P* value
		Odds ratio	95% Confidence interval	
Age	Years	1.02	1.02, 1.03	< 0.001
Sex	Female Male (REF)	0.97	0.85, 1.11	0.657
Occupation	High risk Other (REF)	1.11	0.90, 1.36	0.314
Spatial location				
Coast	Yes No (REF)	1.05	0.91, 1.22	0.475
Rural	Yes No (REF)	1.06	0.92, 1.23	0.384
Urban	Urban associated	1.06	0.84, 1.34	0.614
	Rural Urban (REF)	1.07	0.92, 1.25	0.337
Rural/Inland	Rural coast	1.05	0.84, 1.31	0.644
	Rural inland Urban (REF)	1.07	0.90, 1.27	0.410
Frequency of food consumption				
Meat	High consumption Low consumption (REF)	0.94	0.78, 1.05	0.207
Sausage (Chicken and pork)	High consumption Low consumption (REF)	1.07	0.90, 1.27	0.456
Fish	High consumption Low consumption (REF)	0.94	0.79, 1.12	0.480
Raw vegetables	High consumption Low consumption (REF)	0.91	0.79, 1.05	0.229
Animal contact^†^				
Domestic carnivores	Yes No (REF)	1.05	0.87, 1.26	0.629
Farm animals	Yes No(REF)	0.84	0.52, 1.32	0.447

^†^Animal contact analysis was performed on a subset of the baseline sample. Only participants who responded in the follow-up period were included (n = 2472).

All logistic regression models were adjusted for sex and age.

### Quantile regression results

All quantile regression models were analyzed using the 80th percentile. Therefore the estimates reflect the difference in anti-HEV IgG titers in the 80^th^ percentile of the distribution by the corresponding risk factor assessed. Anti-HEV IgG titers had a median value of 4.3 U/ml (IQR 2.34–27.7 U/ml) and an 80^th^ percentile of 39.3 U/ml (95% CI 36.4–42.8 U/ml).

First, we consider models without spatial location: Age in years was associated with anti-HEV IgG titers (1.1 U/ml; 95% CI 0.9, 1.2; *P* < 0.001) but none of the other considered risk factors were significantly associated (Table 4). Participants who lived in urban associated settings had 14.1 U/ml (95% CI − 1.2, 29.5) higher titers in relation to participants who lived in urban areas, with a trend towards significance (*P* = 0.07) (Table 4).

**Table 4:** Quantile regression analysis on the 80^th^ percentile of risk factors for anti-hepatitis E IgG antibody titers in the SHIP-TREND cohort in Northeast Germany, 2008–2012 (n = 4335).

Risk factor	Category	Quantile regression (Tau 0.8)		
		Estimate (U/ml)	95% Confidence interval	*P* value
Age	Years	1.05	0.90, 1.20	< 0.001
Sex	Female Male (REF)	2.9	− 2.93, 8.72	0.329
Occupation	High risk	3.97	− 6.0, 13.99	0.454
Spatial location	Other (REF)			
Coast	Yes No (REF)	0.45	− 5.65, 6.54	0.889
Rural	Yes No (REF)	3.40	− 3.44, 10.24	0.343
Urban	Urban associated	14.13	− 1.20, 29.46	0.069
	Rural Urban (REF)	4.29	− 2.62, 11.20	0.218
Rural/Inland	Rural coast	2.13	− 7.23, 11.49	0.639
	Rural inland Urban (REF)	3.90	− 4.73, 12.52	0.371
Frequency of food consumption				
Meat	High consumption Low consumption (REF)	− 2.77	− 9.83, 4.29	0.451
Sausage (Chicken and pork)	High consumption Low consumption (REF)	1.02	− 5.37, 7.41	0.753
Fish	High consumption Low consumption (REF)	− 1.52	− 9.06, 6.03	0.717
Raw vegetables	High consumption Low consumption (REF)	− 4.37	− 11.08, 2.35	0.193
Animal contact^†^				
Domestic carnivores	Yes No (REF)	5.79	− 2.48, 14.07	0.178
Farm animals	Yes No (REF)	− 3.44	− 16.98, 10.10	0.641

^†^Animal contact analysis was performed on a subset of the baseline sample. Only participants who responded in the follow-up period were included (n = 2472).

All quantile regression models were adjusted for sex and age.

**Fig. 2:**
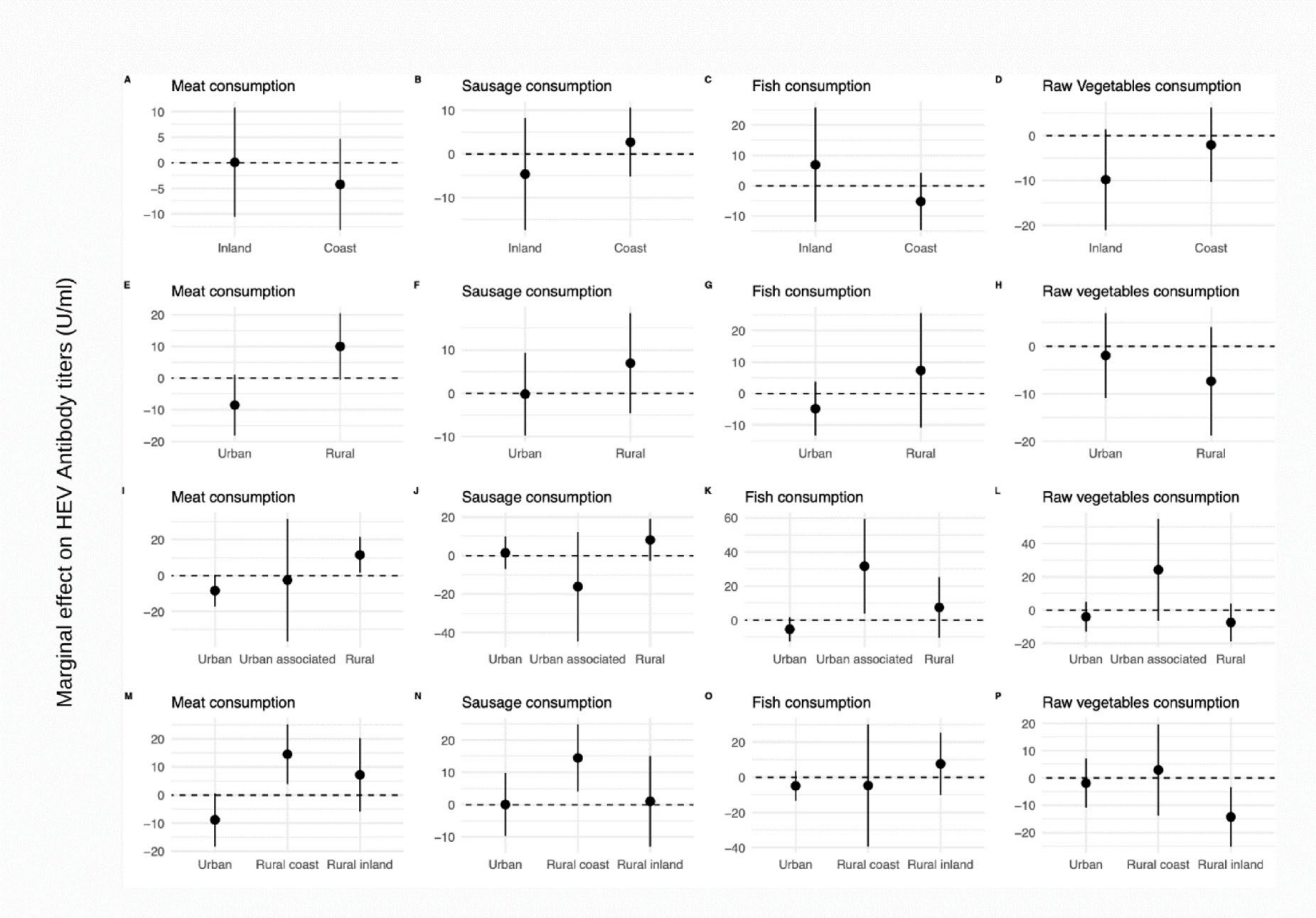
Marginal effects of food groups on anti-HEV IgG antibody titers by spatial location in the SHIP-TREND-0 cohort in Northeast Germany, 2008–2012 (n = 4,335). *Note*: All estimates are computed in relation to the reference category, low consumption for food consumption variables.

When including location as interaction, there were no statistically significant estimates in the inland-coastal regions (Fig. 2A–D). In rural settings participants with high frequency of meat consumption had 10.0 U/ml (95% CI − 0.5, 20.6; *P* = 0.061) higher anti-HEV IgG titer in comparison to those who had low frequency of meat consumption (Fig. 2E). Sausage consumption, fish consumption and raw vegetables had no statistically significant association to anti-HEV IgG in urban/rural locations (Fig. 2F–H). In the model that included the urban/urban associated/rural classification, participants who had a high meat consumption and lived in urban areas had an inverse association, with -8.3 U/ml anti-HEV IgG (95% CI − 17.1, 0.4; *P* = 0.063) (Fig. 2I). Sausage consumption had no statically significant association in this location (Fig. 2J). High fish consumption had a direct association with anti-HEV IgG titers in participants who lived in urban associated areas (31.7 U/ml, 95% CI 3.9, 59.4; *P* = 0.025 (Fig. 2K), and raw vegetable consumption had no statistically significant association in this location (Fig. 2L).

**Fig. 3:**
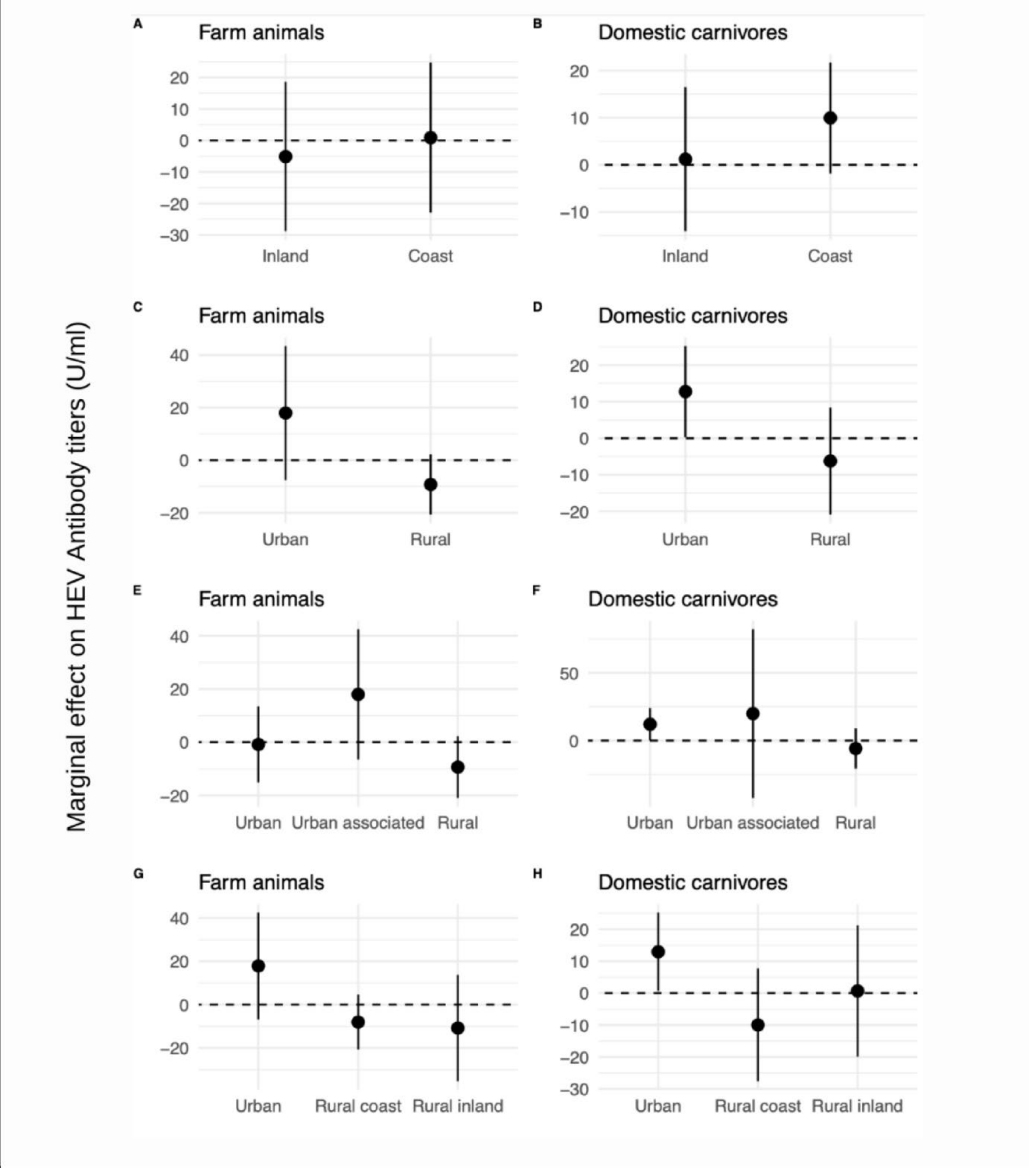
Marginal effects of animal contact on HEV antibody titers by spatial location in the SHIP-TREND-0 cohort in Northeast Germany, 2008–2012 (n = 2,472). *Note*: All estimates were computed in relation to the reference category (no animal contact). Animal contact analysis was performed on a subset of the baseline sample.

Living in rural coastal areas had a similar trend in participants with high meat and sausage consumption, with a significant estimate of 14.5 U/ml (95% CI 3.9, 25.2; *P* = 0.008) and 14.5 U/ml (95% CI 4.1, 24.9; *P* = 0.006), respectively (Fig. 2M,N). Fish consumption, had no statistically significant association in urban/rural coast/rural inland locations (Fig. 2O), but high consumption of raw vegetables in participants who lived rural inland had a significant inverse association with anti-HEV IgG titers in comparison with low consumption, with an estimate of − 14.3 U/ml (95% CI − 25.1, − 3.4; P = 0.01; Fig. 2P). For animal contact, no statistically significant association was found for farm animals (Fig. 3A,C,E,G). Participants that had contact with domestic carnivores in coastal regions had an estimate of 9.9 U/ml (95% CI − 1.8, 21.7; *P* = 0.098 Fig. 3B). However, participants in urban areas who had contact with domestic carnivores had significantly higher titers (12.8 U/ml; 95% CI 0.3, 25.3; *P* = 0.046) than participants without contact. This association was not observed in rural regions (Fig. 3D,F,H).

### Sensitivity analysis

We obtained similar results in the logistic regression analysis, when classifying borderline results as positive. Age in years remained significantly associated with HEV seropositivity (OR 1.03; 95% CI 1.02, 1.03; *P* < 0.001), whilst none of the other risk factors were significantly associated (results not shown).

In the quantile regression models, when computing the estimates across quantiles from the 50^th^ to the 90^th^, we observed a tendency that supports our findings. In the models without spatial location, high meat, fish and raw vegetables consumption had a constant inverse association with anti-HEV IgG titers in all the percentiles, and sausage consumption a direct association (Supplementary Fig. S1). Likewise, high-risk occupation and contact with domestic carnivores maintained a direct association. Contact with farm animals had a constant inverse association with anti-HEV IgG titers (Supplementary Fig. S3). The residential location in the coastal, rural, rural-inland and urban associated areas had a direct association, but rural-coastal areas oscillated between direct and indirect association (Supplementary Fig. S2).

**Fig. 4:**
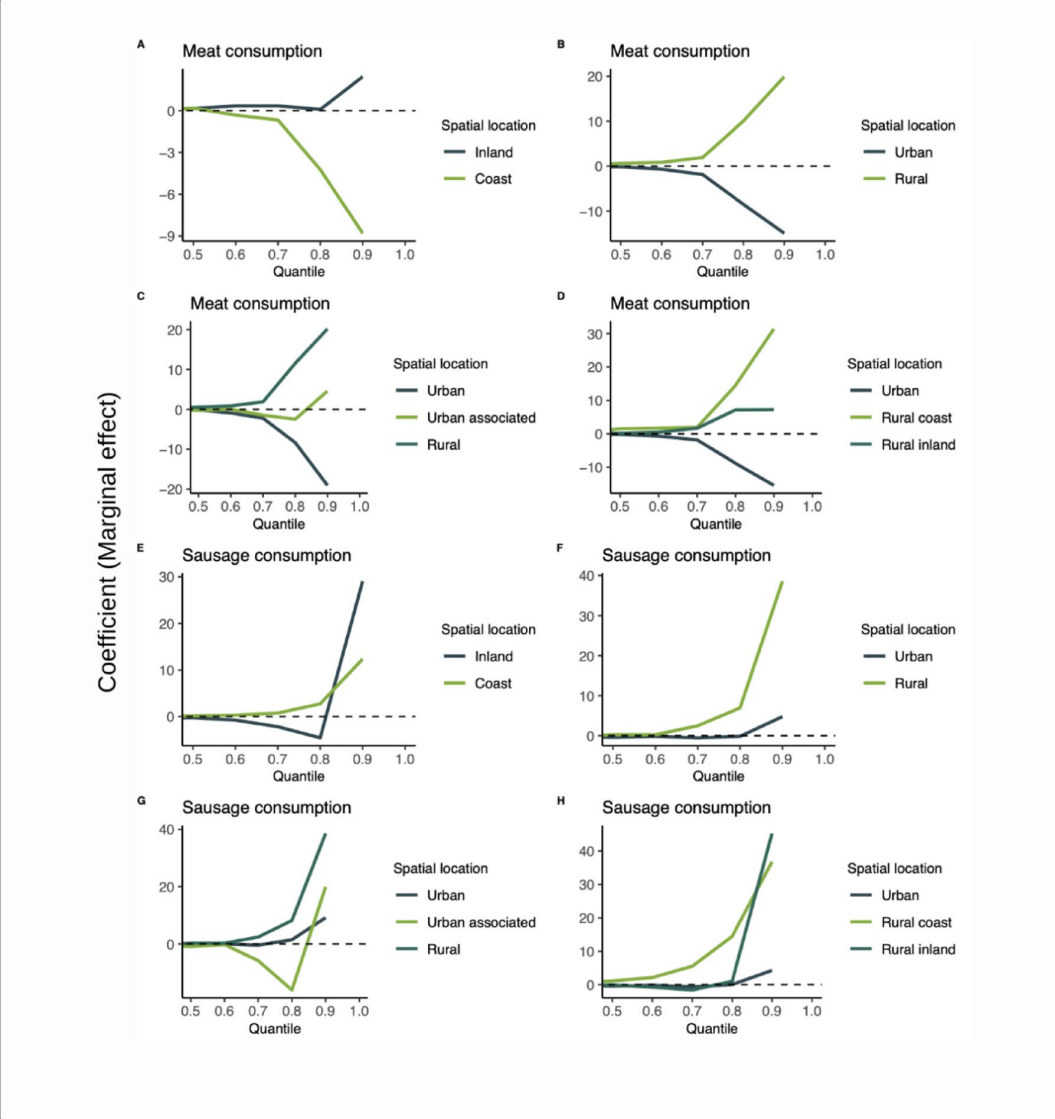
Sensitivity analysis of the marginal effect of meat and sausage consumption on HEV antibody titers by spatial location on SHIP-TREND cohort, 2008–2012 (n = 4,335). Quantile regression from percentile 50^th^ to 90^th^. *Note*: All estimates were computed in contrast with the reference category (low consumption of the food group).

**Fig. 5:**
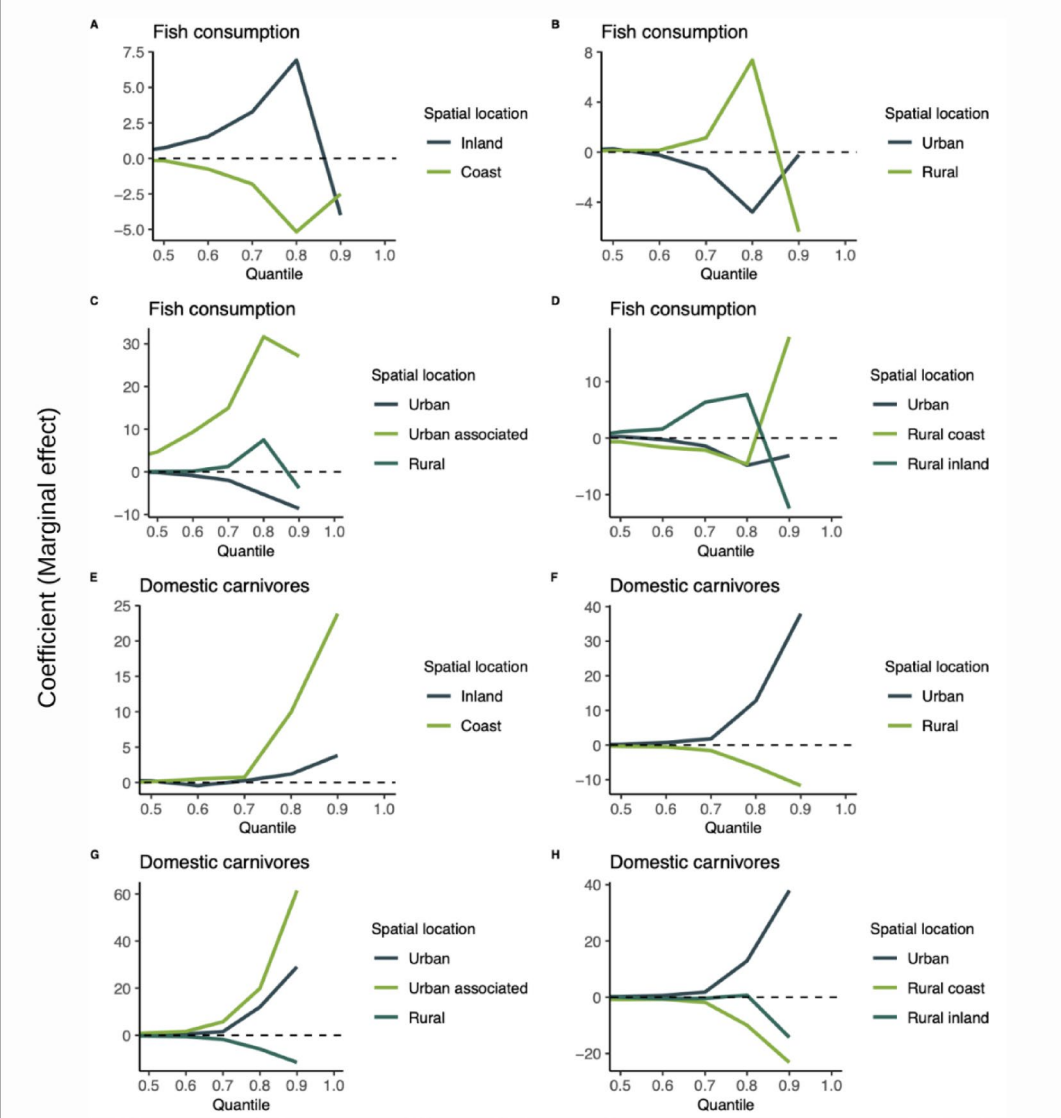
Sensitivity analysis of the marginal effect of fish consumption and contact with domestic animals on anti-hepatitis E IgG antibody titers by spatial location from 50 to 90^th^ percentiles (quantile regression) in the SHIP-TREND cohort, 2008–2012 (n = 4,335). *Note*: All estimates were computed in contrast with the reference category (low consumption of the food group, no contact with the animal group).

In the models that included the spatial component, when computing the estimates across quantiles from the 50^th^ to the 90^th^, we observed that high meat consumption in inland/coastal locations had estimates that were close to zero and maintained a direct trend for inland, and an inverse trend for coastal locations (Fig. 4A). In rural locations high maintained a direct association with anti-HEV IgG titers. In urban locations, the estimate maintained an inverse association (Fig. 4B–D). High sausage consumption had an erratic behaviour in all the spatial locations with estimates oscillating between a direct and inverse effect, except in rural coastal and rural areas, where it kept a direct association with anti-HEV IgG titers (Fig. 4E–H). Similarly, high fish consumption estimates were erratic, except in urban associated areas where it had a direct association with anti-HEV IgG titers (Fig. 5A–D). Raw vegetables had a direct association in urban associated and rural coastal areas and inverse in the other locations (Supplementary Fig. S4A, S4B, S4C, S4D). Contact with domestic carnivores had a consistent behavior in all locations, with a direct association in urban and urban associated areas and inverse in rural areas (Fig. 5E–H). Farm animals had mainly inverse associations, but in some areas the estimates were erratic, changing between direct and inverse in different quantiles (Supplementary Fig. S4E, S4F, S4G, S4H).

## Discussion

Antibody titers are often not correlated with disease severity and protection, but using titers as an outcome might be an interesting alternative to investigate infectious disease exposure ^29^. The design of our study enabled us to assess HEV risk factors outside of dichotomization, regaining the loss in power caused by the transformation of continuous variables into categorical ones ^30^. Furthermore, we could demonstrate that living in rural/urban settings acted as strong effect modifier in our data set. To the authors’ knowledge, this is the first study reporting such an effect modification of risk factors by spatial location on anti-HEV IgG titers.

The strongest effect modification was observed for rural coastal areas in comparison to the other areas types on the effect of sausage consumption and on the effect of meat consumption (Figs. 3, 4). The sensitivity analysis showed a stable trend with positive (+) estimates in all the percentiles analyzed, reflecting the direct association of anti-HEV IgG titers with high meat and sausage consumption in the region. The impact of rural coastal regions might be due to differences in food consumption patterns or the type of meat and sausage consumed, which was not specifically distinguished in SHIP. Pork and wild boar products are considered the primary source of foodborne HEV infection. In 2019, 7.9% of hunted boar specimens and 8.5% of domestic pigs were HEV RNA positive in Mecklenburg-Pomerania ^31^. The region has a high hunting yield in Germany with 65,059 wild boars hunted in 2012/13 (10.1% of Germany’s hunting yield in the period). Although a big proportion of the hunting yield is exported, the consumption of regionally hunted products is popular in the area, especially in rural regions ^32^.

Contact with domestic carnivores was directly related to HEV in urban areas but not in rural areas. The direct association in urban areas was confirmed in the sensitivity analysis, whilst a tendency towards an inverse association was observed in rural areas. In Brandenburg, a bordering state of Mecklenburg-Pomerania, a HEV seroprevalence of 56.6% (95% CI: 46.0, 67.3) in dogs and 32.3% (95% CI: 20.9, 43.75.1) in cats was reported for the period from 2002 to 2005 ^9^. Even though no HEV RNA evidence was detected in that study, there is evidence regarding susceptibility of canine hepatocytes in vitro ^9,33^. The influence of urban areas on the effect of contact with domestic carnivores can be a result of differences in the closeness of animal contact between animal owners and their dogs/cats in urban and rural areas. Pets in urban settings tend to have closer contact with humans because they are more likely to share the living space, whilst dogs and cats in rural settings can spend more time outdoors.

Another potential factor is the increase in popularity of raw-meat based diets for pets in developed countries in recent years ^34^. Nutritional and microbiological risks have been found in these diets due to the presence of a variety of pathogens that can affect the pets and their owners. Moreover, recent evidence of HEV RNA presence in commercial processed pork products opens an inquiry about processed pet food ^34,35^. The risk of raw and processed diets on HEV exposure to dogs, cats and their owners requires further research.

Our findings around meat consumption are in line with other studies in Germany and other European countries, but high sausage consumption had inconsistent results ^6,36^. In the model without spatial location, high sausage consumption was associated with numerically higher HEV antibodies in relation to participants with low consumption, but this association was not significant. When including spatial location in the sensitivity analysis, high sausage consumption in urban associated and inland areas had an inconsistent behavior oscillating between direct and inverse association with anti-HEV IgG titers. This inconsistency might be due to additional processing required for sausage production, the type of meat used or an association between high meat consumption and high sausage consumption in some subgroups.

High fish consumption showed a significant direct association with anti-HEV IgG titers in urban associated areas. In the sensitivity analysis the estimate showed highly erratic behavior when assessed in a sequence of percentiles except in urban associated areas, where it maintained a direct association. No presence of HEV RNA has been detected in fish destined for human consumption to date and no outbreaks have been directly linked to fish consumption but an association has been reported ^6,37,38^. Given that the presence of HEV RNA in surface waters could be a source of contamination for fish/fishermen, this potential exposure requires further research ^39^.

HEV RNA has been detected in fruit and vegetables, but it is unclear if it is an effective source of infection ^6^. We observed in the models without spatial location an inverse non-significant estimate, and a consistent inverse association trend in the sensitivity analysis (Supplementary Fig. S1D). When analyzed with the spatial component, one significant inverse association was found in participants who lived in rural inland areas (− 14.3; CI − 25.1, − 3.4; *P* = 0.01), possibly reflecting lower meat (pork) consumption in participants with high vegetable consumption rather than a protective effect. In the sensitivity analysis, a predominant inverse trend in all locations was observed, except in rural coastal and urban associated areas. In other studies, findings were inconsistent. One study in Germany found that raw vegetables consumption was directly associated with HEV with an OR of 2.0 (95% CI 1.4, 2.8; *P* < 0.03), whilst another study found an inverse association (OR 0.5; 95% CI 0.3, 0.8), and in Italy a direct, but non-significant estimate was obtained ^36,38,40^. No outbreaks or clinical cases have been linked to raw vegetables consumption ^6^. If vegetables are a carrier, it is unclear if contamination takes place during production (e.g. fertilizer) or due to cross-contamination with animal products during food preparation.

Alternative transmission pathways such as direct contact with infected animals, their carcasses or indirect contact with biological animal waste, are thought to have an impact on HEV dynamics. Occupations that expose the individual to contact with potential HEV sources are classified as high risk ^15^. In our sample, participants classified as having a high-risk occupation had a non-significant estimate in relation to those with a low-risk occupation. A tendency towards numerically higher anti-HEV IgG titers was confirmed in the sensitivity analysis, but remained non-significant. In our data set, the percentage of participants with a high-risk occupation was relatively low and comprised a diverse group of occupations. Therefore, our study may have lacked power to identify a significant effect. Participants reporting that livestock or poultry are kept on their property had numerically lower anti-HEV IgG titers, but this association was non-significant and showed inconsistent behavior in the sensitivity analysis. Similar to the high-risk occupation, this inconsistent behavior could be a consequence of a low proportion of participants with exposures to farm animals. Additionally, due to the low number of participants who owned farm animals, we combined the main reservoir species (pigs) with other livestock species such as cattle or small ruminants, which could have introduced noise to the data.

In the period of 2008–2011, the population in the central-north region, where Mecklenburg-Pomerania is located, had a significantly higher HEV seroprevalence (17.7%; 95% CI 15.2, 20.5) than the general German population (15.3%; 95% CI 14.2, 16.5) ^41^. A meta-analysis observed that the estimated seroprevalence from other studies in Germany was dependent on the type of assay used, showing a large variability in the estimated seroprevalence (from 2.60 to 8.50%) ^2^. There is no reference standard for HEV serology, so that assays are compared to another imperfect test in the validation process. Hence, true specificity and sensitivity are unknown ^2,3^. The differences in the estimates can be due to potential measurement error (systematic error) or due to a true difference. Therefore, it is difficult to compare estimates obtained with different anti-HEV IgG titers tests.

Based on our findings, we conclude that the effect of the risk factor on anti-HEV IgG titers in our sample was modified by spatial location. Contact with domestic animals in urban regions compared with no contact has a direct significant association with anti-HEV IgG titers, whilst this association was not observed in rural areas. High meat consumption compared to low consumption in rural regions was directly associated with HEV, but this effect was not observed in urban areas. Quantile regression allowed us to analyze the data with greater detail than logistic regression, capturing estimate behavior beyond the cut-off value. Additionally, we found a higher HEV seroprevalence in the study region compared to the overall German population, but differences may be partly explained by the different assay used. Introducing spatial components in epidemiological research has proved valuable since the beginnings of modern epidemiology ^42^. The interaction of spatial locations with HEV risk factors can be used to enhance evidence-based, solution-oriented interventions that are tailored for the disease dynamic of the study region.

## Supplementary Information

Below is the link to the electronic supplementary material.

Supplementary Material 1

## Data Availability

Data from the “Study of Health of Pomerania” are available from the University Medicine Greifswald, Germany but restrictions apply to the availability of these data, which were used under license for the current study, and so are not publicly available. Data are however available upon reasonable request at https://transfer.ship-med.uni-greifswald.de/FAIRequest/?lang=en and with permission of the University Medicine Greifswald.
