# Supplementary material for: Spatial variation in risk factors for anti-hepatitis E antibody titers in a population-based German study
Source: Sci Rep. 2025 Nov 21;15:41463. doi: 10.1038/s41598-025-26850-z (PMC12644998; doi:10.1038/s41598-025-26850-z)
Supplement: Supplementary file 1 — Supplementary Material 1 [file 41598_2025_26850_MOESM1_ESM.docx]

**Spatial variation in risk factors for anti-Hepatitis E antibody titers in a population-based German study**

**Authors:** Andrea C. Díaz^1, 2, 3 *^, Till Ittermann^1^, Matthias Nauck^4^, Astrid Petersmann^4,5^, Henry Völzke^1^, Birgit Schauer^1^

Supplementary Figures
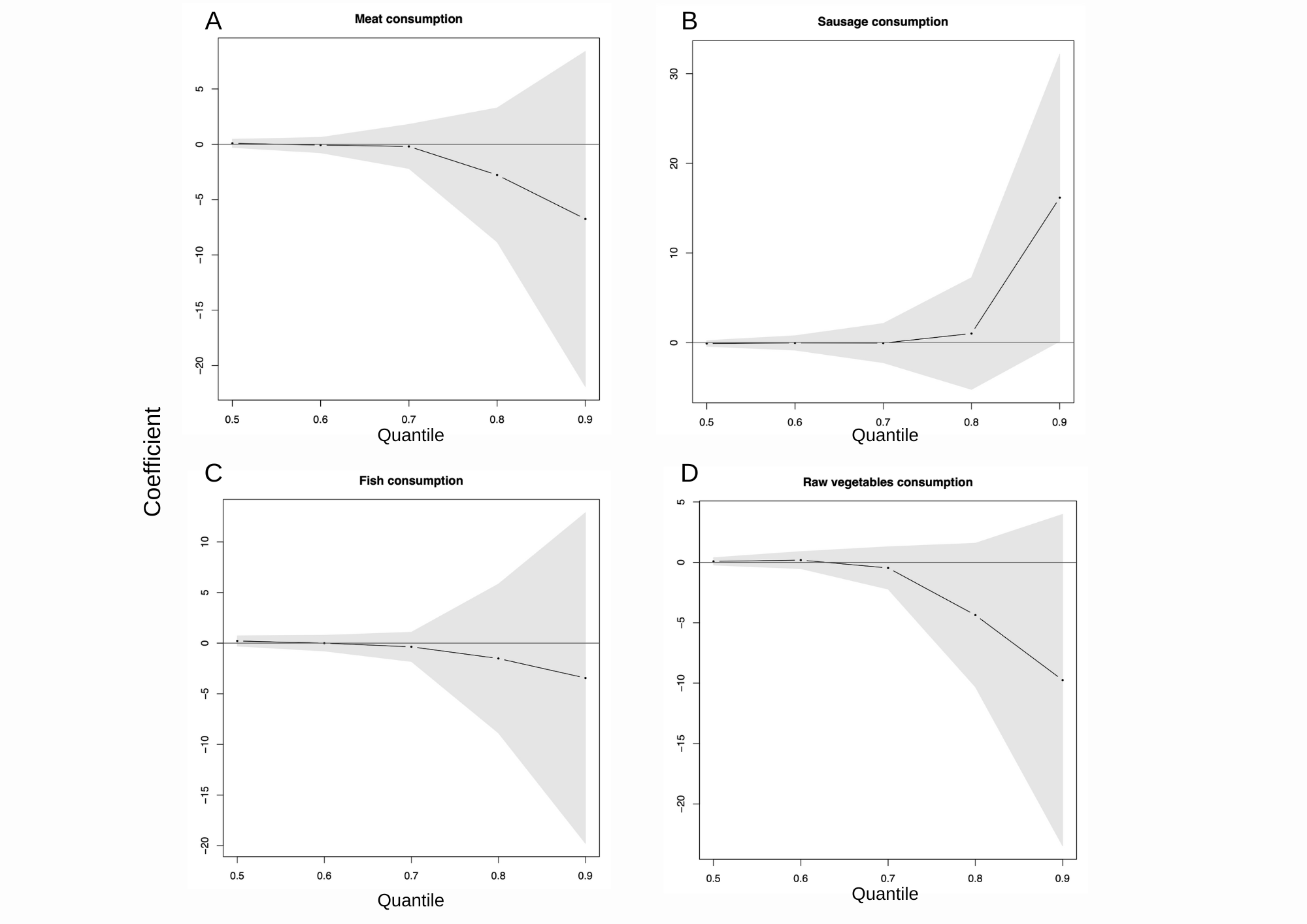


Figure S 1. Sensitivity analysis of the effect of meat, sausage and fish consumption on anti-Hepatitis E IgG antibody titers from 50^th^ to 90^th^ percentiles in the SHIP-TREND cohort in Northeast Germany, 2008-2012 (n = 4,335).

**Note:** All the models are adjusted for sex and age. The estimates are computed in contrast with the reference category (low consumption). Shaded area represents confidence interval of the estimates.


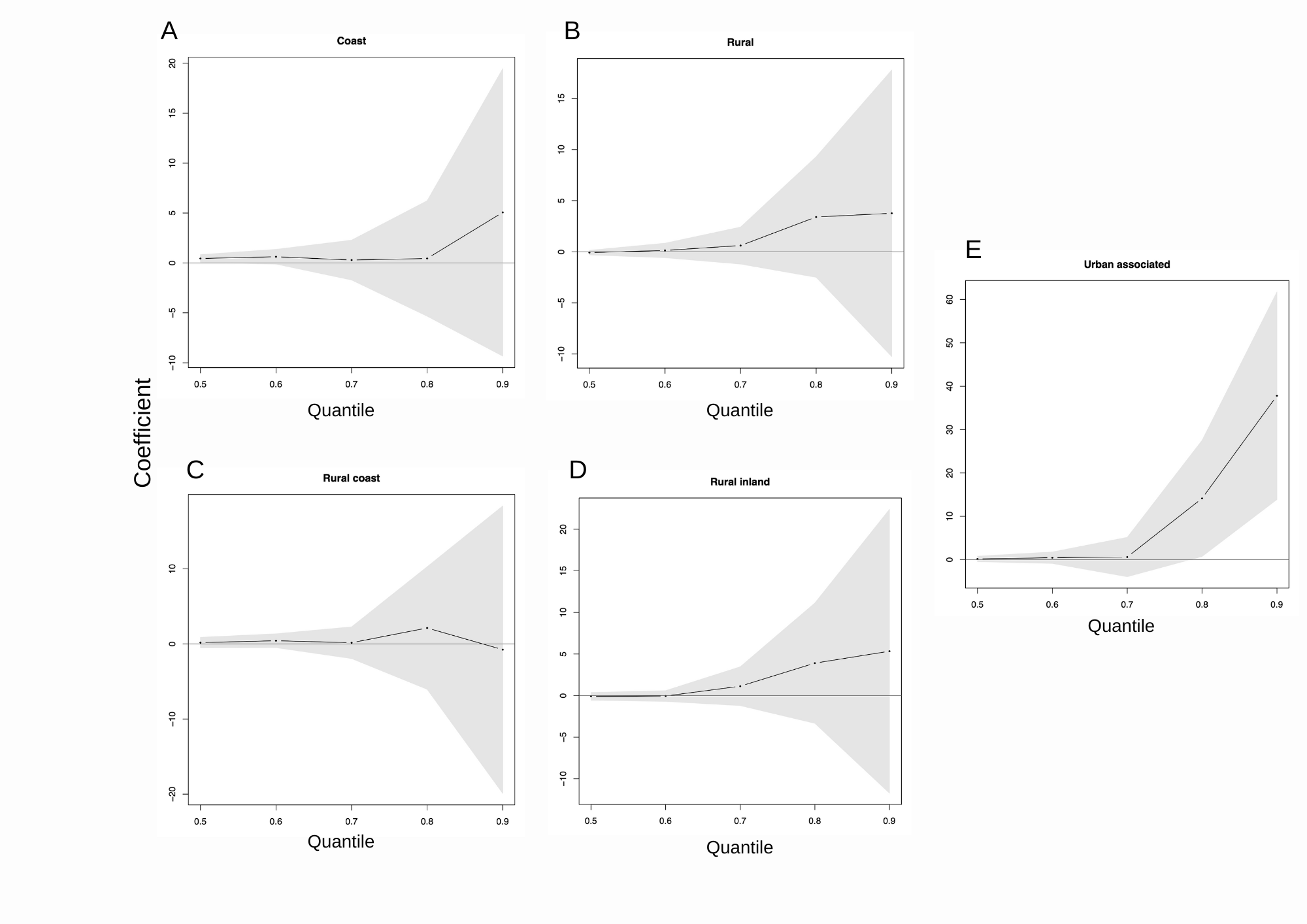


Figure S 2. Sensitivity analysis of the effect of spatial location on anti-Hepatitis E IgG antibody titers from 50^th^ to 90^th^ percentiles in the SHIP-TREND cohort in Northeast Germany, 2008-2012 (n = 4,335).

**Note:** All the models are adjusted for sex and age. The estimates are computed in contrast to the reference category, i.e. urban for rural, urban associated areas, rural coastal and rural inland; and inland for coastal areas. Shaded area represents confidence interval of the estimates.


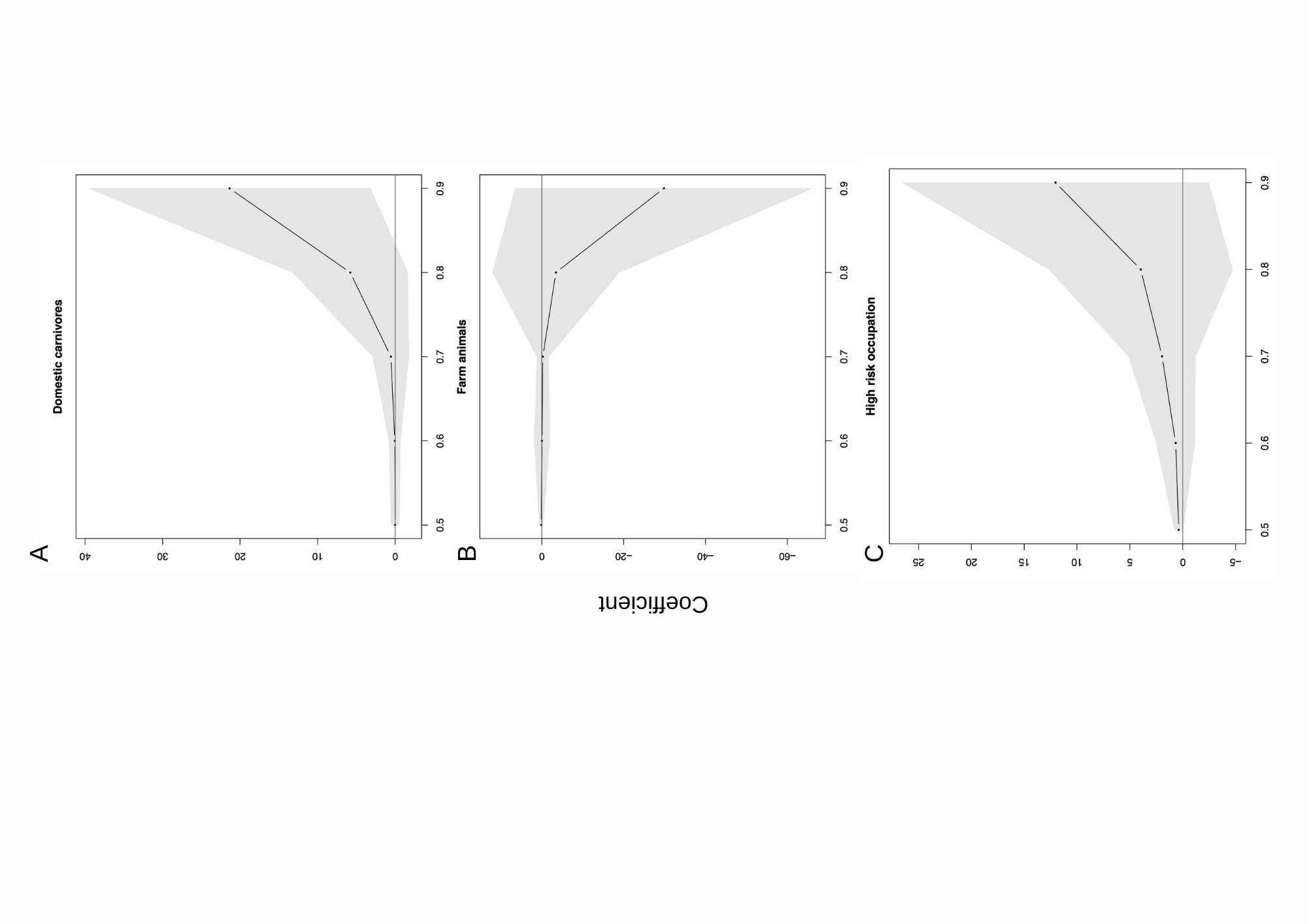


Figure S 3. Sensitivity analysis of the effect of animal contact and high-risk occupation on anti‑Hepatitis E IgG antibody titers from 50^th^ to 90^th^ percentiles in the SHIP-TREND cohort in Northeast Germany, 2008-2012 (n = 4,335).

**Note:** All the models were adjusted for sex and age. The estimates were computed in contrast to the reference category, i.e. low risk occupation and no contact with the animal group. Shaded area represents confidence interval of the estimates. Animal contact analysis was performed on a subset of the baseline sample. Only participants who responded in the follow-up period were included (n = 2,472).


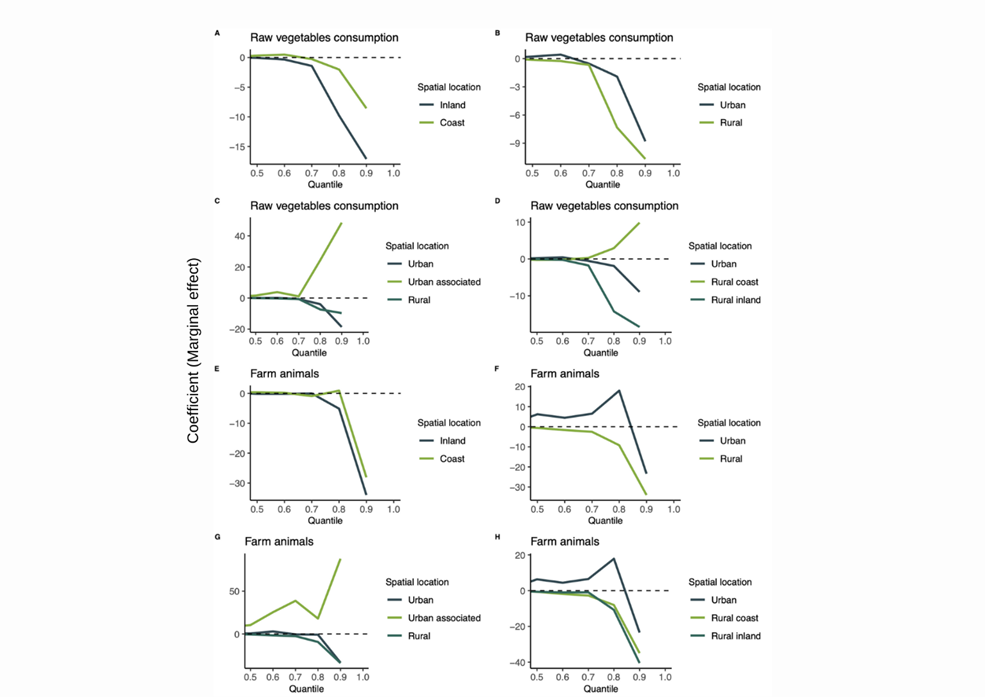


Figure S4. Sensitivity analysis of the marginal effect of raw vegetables consumption and contact with farm animals on anti-Hepatitis E IgG antibody titers by spatial location from 50^th^ to 90^th^ percentiles (quantile regression) in the SHIP-TREND cohort in Northeast Germany, 2008-2012 (n = 4,335).

**Note:** All the models were adjusted for sex and age. All estimates were computed in contrast to the reference category (low consumption of the food group) Animal contact analysis was performed on a subset of the baseline sample. Only participants who responded in the follow-up period were included (n = 2,472).
